# A screening instrument for side dominance in competitive adolescent alpine skiers

**DOI:** 10.3389/fspor.2022.949635

**Published:** 2022-07-22

**Authors:** Maria Westin, Annelie Norlén, Marita L. Harringe, Suzanne Werner

**Affiliations:** ^1^Department of Molecular Medicine and Surgery, Stockholm Sports Trauma Research Center, Karolinska Institutet, Stockholm, Sweden; ^2^Aleris Sports Medicine and Ortopedi, Sabbatsbergs Hospital, Stockholm, Sweden; ^3^The Swedish School of Sports and Health Sciences, Stockholm, Sweden

**Keywords:** alpine skiers, ACL injury, adolescent, dominance, footedness, handedness, ski turn

## Abstract

Previous research has shown that high school ski students injure their left anterior cruciate ligament (ACL) more often than their right ACL, and that a prevention program focusing on equal load to the right and left ski turns prevents ACL injuries. Whether the injuries were in the dominant or non-dominant side of ski students was not determined but may be important knowledge to ski coaches for future design of ski-specific training programs. There is no gold standard on how to investigate the dominant side of alpine skiers. Therefore, the aim of this study was to develop a screening instrument consisting of five questions for identifying side dominance and to evaluate side dominance in competitive adolescent alpine skiers. First, 121 competitive adolescent alpine skiers answered the questions on side dominance using a test-retest design. The questions were: which hand/arm (left/right) or foot/leg (left/right) one uses as the first choice when writing, throwing, kicking a ball, jumping over a fence and stair-climbing. A question about safer/better ski turn to the left or to the right was also added. Second, 274 skiers answered the questions at one occasion. A very good agreement was shown in writing and throwing and kicking a ball, and a fair agreement was shown in jumping over a fence and stair climbing. A total of 243 skiers reported right-sided dominance, and seven skiers reported left-sided dominance. One hundred and nineteen of the 121 skiers who took part in the test-retest design answered the question safer/better ski turn, and of those 70 (59%) reported that they had a safer/better ski turn to one side than to the other side. However, the side was not consistent between the two test occasions, and the question did not correlate with side dominance. A combination of the three questions “What hand/arm do you use as first choice when writing?” “What hand/arm do you use as first choice when throwing?” and “What foot/leg do you use as first choice when kicking a ball?”, may be used to decide side dominance in adolescent alpine skiers. Most adolescent alpine skiers reported right-sided dominance.

## Introduction

Dominant vs. non-dominant side is commonly discussed when it comes to physical performance and injury risk factors in different sports. However, the definition of the dominant and non-dominant sides often varies between studies.

Hewett et al. (1996, 2001) defined leg dominance as a side-to-side difference in muscle strength and coordination, where the dominant leg is superior to the non-dominant leg for both muscle strength and coordination. Herring (1993) defined dominance as the ability of an individual to perform a certain task with greater accuracy, speed, and agility with the dominant leg than with the non-dominant leg. Ross et al. (2004) reported better knee joint proprioception, thigh muscle strength, and knee flexion range of motion of the dominant leg than the non-dominant leg. Moreover, a dominant or non-dominant side is less evident in terms of the lower extremities than the upper extremities, meaning that a side-to-side difference is much more common in the upper extremities than in the lower extremities (Gabbard, 1993). In the general population, the prevalence of right-leg dominance varies between 75 and 93% (Didia and Nyenwe, 1988; Gabbard, 1993; Gabbard and Iteya, 1996; Schneiders et al., 2010; Ziyagil, 2011; Steidl-Müller et al., 2018). The corresponding number for the upper extremities is 89–96% (Chapman and Chapman, 1987; Ziyagil, 2011).

Several studies on the area of sports medicine have defined dominant leg to be the leg that one chooses when kicking a ball (Ross et al., 2004; Negrete et al., 2007; Brophy et al., 2010; Ruedl et al., 2012). The most commonly used tests are kicking a ball, unilateral balance with eyes closed, and hopping. These tests are thought to evaluate both muscle force and joint stability of the lower extremities (Gundersen et al., 1989; Herring, 1993). A side-to-side difference can increase injury risk in either the dominant or the non-dominant leg (Hewett et al., 2010). For example, the individual may rely too much on the dominant leg and thereby injure that leg, or the non-dominant leg may be injured due to decreased muscle strength and impaired coordination (Hewett et al., 2010).

Leg dominance has been discussed as a potential risk factor for sustaining an anterior cruciate ligament (ACL) injury (Matava et al., 2002; Negrete et al., 2007; Brophy et al., 2010; Ruedl et al., 2012). Negrete et al. (2007) studied the relationship among leg dominance, side of injury, and gender on 302 subjects with non-contact ACL injuries. Both genders injured the dominant as well as the non-dominant leg, and men and women did not differ in this aspect. However, they found a strong tendency for the women to injure their left knee compared to their right knee. This was also found by Brophy et al. (2010) who reported that the left knee was injured in 68% of female soccer players and 26% of male soccer players with non-contact ACL injuries. They found that male soccer players more often injured their kicking leg (dominant leg) than their supporting leg (non-dominant leg), whereas female soccer players predominantly injured their supporting leg (non- dominant leg). In another study (Matava et al., 2002), the dominant knee was not found to be a risk factor for sustaining a non-contact ACL injury. However, in a study on 143 German female soccer players the dominant leg was found to be more injury-prone regarding overuse and contact injuries (Faude et al., 2006).

The incidence of injuries in both recreational and competitive alpine skiers is high, and most injuries are related to the lower extremities. Whether the injuries occur in the dominant or non-dominant side is rarely reported. In an epidemiological study during 25 ski seasons, no side differences were found investigating ACL injuries (Pujol et al., 2007). In another study on recreational skiers, it was shown that 63% of ACL injuries were to the left knee (Urabe et al., 2002), and that female alpine skiers had twice as high risk to sustain an ACL injury in their non-dominant leg compared to their dominant leg (Urabe et al., 2002; Ruedl et al., 2012). Westin et al. (2012) showed that it was a higher risk to sustain an ACL injury in the left than in the right knee in adolescent alpine skiers (Westin et al., 2012, 2018). Whether this was in the dominant or non-dominant side was not determined.

Majority of publications in sports medicine define side dominance using only one question (Herring, 1993; Faude et al., 2006; Negrete et al., 2007; Brophy et al., 2010; Ruedl et al., 2012). Side dominance is often used to find out whether a functional movement is performed better on one leg compared to the other. However, functional hop tests are complex and challenge the whole body. Therefore, we find it important to take into account both the lower and upper extremities. Moreover, it is likely to be believed that using only one question may not be sufficient to determine side dominance.

The aim of this investigation was to develop a screening instrument consisting of five questions regarding side dominance in competitive adolescent alpine skiers. The second aim was to conduct a survey identifying side dominance, and the third aim was to study whether competitive adolescent alpine skiers had a safer/better ski turn to the left or to the right and if this was correlated with side dominance.

## Methods

### Design and ethical approval

A test-retest design to develop a screening instrument for dominance and a cross-sectional design to determine dominance in competitive adolescent alpine skiers were used in 2012. The study was approved by the Swedish Ethical Review Authority (Dnr 2006/833-31/1). Oral and written consent was collected from all participating skiers.

### Subjects

All competitive adolescent alpine skiers studying in one of the Swedish ski high schools during the spring and/or the autumn 2012 were asked to participate in the study. In the first test occasion (spring 2012), 252 skiers were invited, and 198 accepted the invitation. In the second test occasion (autumn 2012), 241 skiers were invited and 197 accepted the invitation.

A total of 274 competitive adolescent alpine skiers (130 men, mean age 17.8 ± 1.3 years, and 144 women, mean age 17.7 ± 1.4 years) accepted to participate in the study. Of these, 121 competitive alpine skiers (59 men/62 women) were included in a test-retest design and answered the screening instrument in two occasions with approximately 4 months in-between the tests.

### Screening instrument

The screening instrument consisted of five questions about side dominance and was obtained from earlier publications (Chapman and Chapman, 1987; Chapman et al., 1987; Didia and Nyenwe, 1988; Elias et al., 1998; Annett, 2000). Two questions focused on the upper extremities: “which hand do you prefer to write with (writing) and which arm do you prefer to throw with (throwing)?”, while three questions focused on the lower extremities: “which foot do you prefer to kick a ball with (kicking), which leg do you prefer to lift first when jumping over a fence (jumping), and which leg do you prefer to lift first when climbing a stair (climbing)?”. In addition, there was a sport-specific question: “do you have a safer/better ski turn to the left or to the right?”.

### Procedure

Each skier answered the questions under the supervision of the principle investigator (MW). Skiers who were uncertain about how to answer were allowed to practice the tasks before giving the answer.

### Statistical analyses

Descriptive data are presented with mean ± SD and frequencies and percentages. Cohen's kappa analysis was conducted to assess the test-retest reliability of the questions. The results were interpreted according to the recommendation by Landis and Koch (1977) (0.01–0.2 poor agreement, 0.21–0.4 fair agreement, 0.41–0.6 moderate agreement, 0.61–0.8 substantial agreement, and 0.81–1 almost perfect/excellent). Percentage agreement was also calculated to evaluate the agreement between the two test occasions. An explorative factor analysis was completed to explain the variability between the sets of tasks. In cases where a skier had not answered a question, the question was excluded as missing, while the other questions were included in the survey of dominance. Chi-2 square test was conducted to evaluate differences between male and female skiers. Level of statistical significance was set at *p* < 0.05. All statistical tests were processed using STATISTICA 10.0.

## Results

### Test-retest reliability

A total of 121 subjects participated in the test-retest of the questions. The results of the test-retest showed a kappa of 1 for the three questions about writing, throwing, and kicking a ball. The questions about jumping over a fence and stair climbing showed a kappa of 0.4 and 0.22, respectively (Table 1).

**Table 1:** Kappa coefficient, which is the quantity of agreement, adjusted for chance, and percentage of agreement between the first and second test occasions.

	**Answers**				**Kappa analysis**	
**Task**	**(*n*)**	**Left (*n*)**	**Right (*n*)**	**Ambivalent (*n*)**	**Agreement (%)**	**Kappa coefficient**
Writing	121	5	116	0	100	1.0
Throwing	121	3	118	0	100	1.0
Kicking	118	6	112	0	100	1.0
Jumping	119	19	71	19	75.6	0.4
Climbing	114	10	73	31	72.8	0.22

The exploratory factor analysis demonstrated that writing, throwing, kicking a ball, jumping over a fence, and stair-climbing were loaded on two factors in both test occasions. In the first test occasion, the two factors explained 54% of the variance and in the second test occasion 57%. Writing throwing, and kicking a ball were loaded to one factor, and jumping over a fence and stair-climbing were loaded to another factor. The question about safer/better ski turn did not show any relationship to the other five questions (Table 2).

**Table 2:** Exploratory factor analysis on the first and second test occasions.

	**Test occasion 1**				**Test occasion 2**			
**Task**	**Factor 1**		**Factor 2**		**Factor 1**		**Factor 2**	
Writing	0.82^*^			0.07	0.86^*^			0.04
Throwing	0.87^*^			0.05	0.87^*^			0.18
Kicking	0.80^*^			0.01	0.73^*^			−0.09
Jumping		0.12	0.69^*^			0.14	0.81^*^	
Climbing		0.02	0.75^*^			0.00	0.83^*^	
Ski turn		0.01	−0.38			0.02	−0.10	

* p < 0.05.

### Survey of side dominance

The questions about jumping over a fence and stair-climbing showed a fair agreement and were therefore excluded from the survey. Consequently, side dominance was defined when the skier gave the same answer, left or right, on the following three questions about writing, throwing, and kicking a ball.

Out of the 274 skiers, 243 reported right-sided dominance regarding the questions writing, throwing, and kicking a ball, while seven reported left-sided dominance (Table 3). In total, 253 of the skiers (92%) preferred to use the right hand when writing, 260 (96%) preferred to throw with the right arm, and 253 (94%) chose the right leg for kicking a ball (Table 3). The female skiers showed a higher percentage of matched answers than the male skiers (Table 3). The female skiers were, to a greater extent, right-dominant regarding each separate question (Table 3).

**Table 3:** Distribution of the answers about dominant/non-dominant side for each question.

	**Male skiers (*****n*** = **130)**			**Female skiers (*****n*** = **144)**			**All skiers (*****n*** = **274)**		
**Task**	**Left** ***n*** **(%)**	**Right** ***n*** **(%)**	**Drop-out** **(*****n*****)**	**Left** ***n*** **(%)**	**Right** ***n*** **(%)**	**Drop-out** **(*****n*****)**	**Left** ***n*** **(%)**	**Right** ***n*** **(%)**	**Drop-out** **(*****n*****)**
Writing	14 (11)	116 (89)	0	7 (5)	135 (95)	0	21 (8)	253 (92)	0
Throwing	9 (7)	121 (93)	0	3 (2)	139 (98)	2	12 (4)	260 (96)	2
Kicking a ball	10 (8)	117 (92)	3	7 (5)	136 (95)	1	17 (6)	253 (94)	4

### Ski turn

One hundred and nineteen of skiers answered the question about safer/better ski turn twice. Out of these, 70 answered a safer/better ski turn in one direction, 27 answered that they did not have a safer/better ski turn, and 22 reported different answers in the first and second test occasions. A total of 97 matched answers (kappa 0.58) were judged to be a moderate agreement. Of the 70 skiers that reported to have a safer/better ski turn, 47 answered the same direction in both test occasions, 30 answered the left turn, 17 the right turn, and 20 were uncertain. Three skiers did not answer direction (left/right) the second time and thus were treated as dropouts. No gender differences were shown regarding the answers about ski turn.

Sixteen out of the 17 skiers who answered a safer/better ski turn to the right in both test occasions were also categorized as right-side dominant, meaning they preferred to write with the right hand, throw with the right arm, and kick a ball with the right foot (kappa 1). One skier preferred to use the left hand when writing, throwing, and kicking a ball. Twenty-seven out of the 30 skiers who answered a better/ safer ski turn to the left reported right-sided dominance, and three answered the right side in two of the three questions. No kappa analysis could be conducted because of the absence of left-side dominance. Eighteen out of the 20 skiers who were uncertain reported right-side dominance and one left-side dominance (kappa 0.95). One skier answered right side in two of the three questions.

Seventy out of the 153 skiers who only answered the question once reported a safer/better ski turn to the left, 54 to the right, and 28 did not report to have any safer/better direction of their ski turn.

## Discussion

The overall aim of the present investigation was to develop a screening instrument for identification of dominant/nondominant side in adolescent competitive alpine skiers. The tasks writing, throwing, and kicking a ball showed an almost perfect agreement, while the tasks jumping over a fence and climbing a stair showed a fair agreement. Despite the moderate agreement demonstrated in the questions about jumping over a fence and the foot one prefers to use when climbing a stair, the kappa coefficient was low. This can be explained by the fact that the kappa coefficient is high if the outcomes of various alternatives are approximately equal (Feinstein and Cicchetti, 1990). The female skiers reported a higher number of matched answers than the male skiers, which is in accordance with the previous research by Schneiders et al. (2010). The second aim of this study was to determine whether the skiers were right- or left-side dominant. In the survey, the tasks jumping over a fence and climbing a stair were excluded because of low kappa values in the reliability study. The result of the survey showed that majority of the skiers reported the right side to be the dominant side (89%). This is in line with the general population (Didia and Nyenwe, 1988; Gabbard, 1993; Gabbard and Iteya, 1996; Schneiders et al., 2010; Ziyagil, 2011) and higher than previously published when using several questions (Chapman and Chapman, 1987; Chapman et al., 1987; Didia and Nyenwe, 1988; Friberg and Kvist, 1988; Gabbard, 1993; Gabbard and Iteya, 1996) or solely one question (Brophy et al., 2010; Ruedl et al., 2012).

In the literature there are a number of quantitative assessment scores reporting good reliability in terms of side dominance of the arm/hand such as the Edinburgh Handedness Inventory (Oldfield, 1971) and Chapman and Chapman (1987) measurement of handedness. Both these scores include a large number of tasks that may jeopardize the compliance in answering the questions. Therefore, we chose solely five questions in order to assess side dominance. In accordance with the Edinburgh Handedness Inventory (Oldfield, 1971) and the measurement of handedness by Chapman and Chapman (1987), our survey included the tasks writing and throwing. A recent publication with a short form of the Edinburgh Handedness Inventory including these questions was found to have a good reliability (Veale, 2014). Schneiders et al. (2010) conducted a reliability study that included 12 different lower limb performance tasks and found that kicking a ball had a substantial agreement, and that climbing a stair had a fair agreement (Schneiders et al., 2010). They also found that self-rated side dominance of the lower extremities was only correlated with the tasks kicking a ball and picking up a marble (Schneiders et al., 2010).

In the present study the factor analysis showed that writing, throwing and kicking a ball were loaded to one factor a nd the question jumping over a fence and climbing a stair were loaded to another factor. Schneiders et al. (2010) also found that kicking a ball and stair-climbing were loaded to different factors. One might speculate that kicking and throwing a ball are something that a child starts with quiet early in life and therefore is not influenced by the surrounding environment when choosing which hand or foot to use. We know that the cerebral hemispheres are asymmetric (Steinmetz, 1996), and that individuals almost always choose one side over the other (Gabbard, 1993; Gabbard and Iteya, 1996). However, previous literature has shown that children in general are two-footed until around the age of 11 years old and thereafter become more or less lateralized (Gabbard and Iteya, 1996). Consequently, a more complex skill such as alpine skiing may be influenced by side preferences but possibly also how skills are learned and thus not necessarily guide us to understand laterality.

In a study on competitive alpine skiers, Westin et al. (2012) reported that two-thirds of lower extremity injuries occurred in the left leg. Based on this information, the question on safer/better ski turn was formulated. All the skiers, except for one who reported to have a safer/better ski turn, were classified as right-side dominant according to our three questions, although no correlation between side dominance and ski turn was found. Twenty percent of the skiers in the test-retest were sure of having a safer/better ski turn but were unsure of what direction, left or right. A possible reason for this uncertainty could be the time of the test occasions, in the beginning and at the end of the preseason, when the skiers had not yet been on snow. Another reason may be that the direction of a safer/ better ski may be more of a learned skill than a native skill. Whether knowledge of side dominance is helpful in understanding the direction of a safer/better ski turn is not known and can be questioned if the ski turn is altered between test occasions. However, since skiing is an equilateral sport, awareness of dominance may be helpful knowledge in the training phase of alpine skiing. According to Hewett et al. (2010), the non-dominant side lacks strength and coordination compared to the dominant side and thereby possibly needs more attention. When implementing a prevention program focusing on the skier's ability to perform neuromuscular exercises equally good on both legs, it could be helpful for the skier to understand where his or her weaknesses lie. To highlight dominance may therefore be of further help during training to become equally good in both directions in alpine skiing.

This study included a homogenous group of adolescent competitive alpine skiers and can therefore only be generalized to young alpine skiers. The same investigator supervised both test occasions ensuring the same instructions was given, which could be regarded as a study strength. The time of 4 months between the two test occasions may reduce possible recall bias, although side dominance is suggested to be stable after the age of 11 years (Gabbard and Iteya, 1996).

## Conclusion

The reliability test of side dominance showed an almost perfect agreement in the tasks writing, throwing, and kicking a ball. Using three questions, the alpine ski students reported right-side dominance. There was a considerable uncertainty among the alpine skiers whether they had a safer/better ski turn to the left or to the right, and this was not correlated with side dominance.

## Data availability statement

The raw data supporting the conclusions of this article will be made available by the authors, without undue reservation.

## Author contributions

MW and SW have given substantial contribution to the conception and design of the study. Acquisition of data by AN, and analysis and interpretation by MW, AN, MH, and SW. The manuscript was drafted by MW. All authors critically revised, read, and approved the final version of the manuscript.

## Conflict of interest

The authors declare that the research was conducted in the absence of any commercial or financial relationships that could be construed as a potential conflict of interest.

## Publisher's note

All claims expressed in this article are solely those of the authors and do not necessarily represent those of their affiliated organizations, or those of the publisher, the editors and the reviewers. Any product that may be evaluated in this article, or claim that may be made by its manufacturer, is not guaranteed or endorsed by the publisher.
